# Factors Associated with the Willingness to Become a Living Kidney Donor: A National Cross-Sectional Study

**DOI:** 10.3390/ijerph19031313

**Published:** 2022-01-25

**Authors:** Paulina Kurleto, Lucyna Tomaszek, Irena Milaniak, Wioletta Mędrzycka-Dąbrowska

**Affiliations:** 1 Faculty of Medicine and Health Sciences, Andrzej Frycz Modrzewski Krakow University, 30-705 Cracow, Poland; pkurleto@afm.edu.pl (P.K.); ltomaszek@afm.edu.pl (L.T.); imilaniak@afm.edu.pl (I.M.); 2 Department of Pneumonology and Cystic Fibrosis, National Institute of Tuberculosis and Lung Disorders, 34-700 Rabka-Zdrój, Poland; 3 Department of Anesthesiology Nursing and Intensive Care, Medical University of Gdansk, 80-211 Gdansk, Poland

**Keywords:** living kidney donation, attitude, life satisfaction, personal values, factors

## Abstract

Introduction: Living donor kidney transplantation is the preferred method of treating kidney failure. The donor agrees to undergo an elective procedure for the benefit of the recipient. Aim: To assess the attitude toward living kidney donation and to investigate the factors that contribute to kidney donation willingness. Methods: A cross-sectional study was carried out between December 2020 and February 2021. The study covered a representative group of 953 Poles aged 18−77, living in all Polish voivodships. The relationship between sociodemographic factors, personal values (Personal Values List), the total score of life satisfaction (Satisfaction with Life Scale) and the willingness to donate a kidney to another human was assessed using a logistic regression model. Results: The most frequently chosen personal values were: good health; physical and mental fitness; love and friendship; knowledge and wisdom. The most frequently chosen symbols of happiness were: good health, successful family life, being needed by others. The median satisfaction with life for the entire group was 20 [16; 24]. Voluntary donation of a kidney to another human being i.e., family, friends, strangers were more often declared by women (OR = 1.21; Cl95%: 1.03−1.42), for whom the most important symbol of happiness was a life full of adventures, travels (OR = 1.39; Cl95%: 1.06−1.82) and the most important personal value was goodness and tenderness (OR = 1.21; Cl95%: 1.05−1.40). Total scores of The Satisfaction with Life Scale correlated positively with the willingness to voluntarily donate a kidney (OR = 1.03; Cl95%: 1.003−1.06), while age correlated negatively (OR = 0.99; Cl95%: 0.98−0.99). Conclusions: Respondents who declare their willingness to be a living kidney donor are mainly female, for which the most important symbol of happiness is a life full of adventures and travel, and the most important values are personal goodness and tenderness. The desire to donate a kidney to another person decreases with age and grows with life satisfaction. Trial registration: ClinicalTrials.gov (ID: NCT04789122).

## 1. Introduction 

Living donor kidney transplantation is the preferred method of treating kidney failure because it results in longer graft survival and better life quality than chronic dialysis or deceased donor kidney transplantation [1]. Unfortunately, the demand for kidney transplantation still exceeds the availability of this organ worldwide. In Eastern and Central European countries, functioning kidney transplant patients account for less than a third of all patients treated for chronic kidney disease or renal failure [2]. Therefore, increasing donation rates requires the intensification of educational activities related to acquiring kidneys from living donors, especially among family and friends of the recipients [3], unrelated persons, and as part of the paired exchange programs [4].

Living kidney donation has been practiced in Poland since 1966 [5]; however, it accounts for a low percentage of all kidney transplants performed. From the beginning of the register of living organ donors—that is, from 1 January 2007 to 31 December 2020—608 living kidney donors have been registered. In 2020, 2,101 patients with an average age of 46 years and 7 months were waiting for a kidney transplant in Poland. The most living kidney transplants were performed in 2015 (*n* = 60). In 2016−2019, the average annual number of transplants was around 50, while in 2020 it was only 31. The decline in transplant rates should be related to the reduced activity of live donation coordinators due to the SARS-COV−2 pandemic; the live organ donation program was suspended for most months [6]. These numbers, with the population of Poland counting around 37.8 million in 2020, indicate much smaller options for treating patients with this method compared to other European countries, such as Spain (259 donors/46.8 mln inhabitants), Italy (284 donors/60 mln inhabitants), the Netherlands (367 donors/17.1 mln inhabitants) [7]. According to Hermanowicz et al., this low rate may be due to lack of national and high quality systems in the living kidney donation field [8]. 

In Poland, kidney transplantation is regulated by the *Act on the collection*, *storage and transplantation of cells*, *tissues and organs of 2005*. A living kidney donor may be: a direct relative in a straight line; siblings; a spouse and a non-relative, if it is justified by emotional or personal reasons—which, in this particular case, the consent of the court and the opinion of the Bioethics Committee are required. The Act also allows cross-kidney transplantation [9], but does not allow organ transplants from unrelated donors, unlike laws in some countries that have legalized donation to a stranger [10,11,12,13,14,15,16]. 

The willingness to become a living donor depends both on who the kidney will be donated to, and the feeling of safety of the potential donor. People who consider becoming a kidney donor must carefully weigh the potential risks and benefits of donating a kidney. In the decision-making process, factors such as medical risk, awareness of the consequences of kidney removal, readiness for convalescence or the inability to immediately return to work are important [17]. According to recent studies [4,11,16,18], sociodemographic factors may also have an impact on the willingness to donate an organ (for instance: age, gender, religious, race/ethnicity, income, education level, marital status).

It also seems that personal values and level of life satisfaction may be related to the willingness to donate a kidney.

The value system that people have regulates their choices and behavior and serves to express what is especially dear to them. Values, therefore, constitute the regulator of conscious and purposeful action in various life situations and decisions. One’s values and belief system have motivational power and are a guide for an individual’s choices of action. These include the decision regarding becoming a potential living donor [19]. In the field of organ transplantation, especially in living donation, the roles of altruism, empathy and personal values are significant to the well-being of society [20,21].

Satisfaction is a positive emotional state that is experienced as a result of achieving a desired goal [22]. The available survey provides input about the life satisfaction index among living organ donors. The study suggests that living kidney donors report satisfaction with their lives on par with or greater than the general population. Also, donation experiences were positively related to satisfaction with life [23].

However, to our best knowledge, no studies on the connection between life satisfaction and personal values and the willingness to donate have been found in the latest literature. It also seems important to take a closer look at the value system of potential kidney donors, as they often determine their choices and behaviors. A better understanding of the attitudes towards living kidney donation among the general public is important when conducting public health and educational programs to support this type of treatment.

In view of the above-mentioned considerations, the aim of this study was to assess and investigate the relationship between the sociodemographic factors, personal values, life satisfaction and the willingness to become a living kidney donor among Polish citizens. 

## 2. Materials and Methods

### 2.1. Study Design, Setting

**Figure 1:**
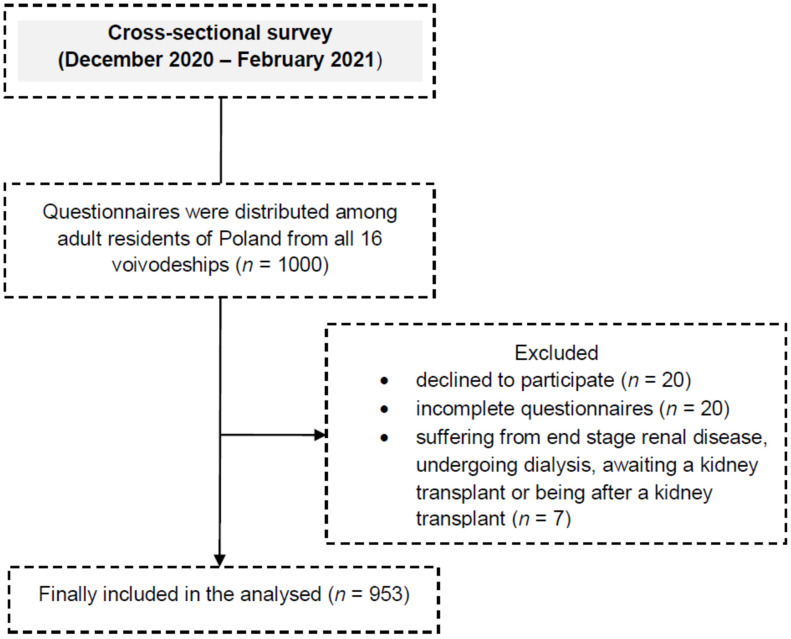
Flow Diagram.

A cross-sectional study was carried out between December 2020 and February 2021 after obtaining the consent of the Bioethics Committee of the Andrzej Frycz Modrzewski Krakow University (decision no. KBKA /51/O/2020). The study included a group of 953 randomly selected adult residents of Poland (Figure 1). The guidelines of the Helsinki Declaration (World Medical Association, 2013) and STROBE (Strengthening the Reporting of Observational Studies in Epidemiology) [24], as well as The General Data Protection Regulation [25] were followed. The study was registered at ClinicalTrials.gov (ID: NCT04789122). 

### 2.2. Participants 

People of both sexes, over 18 years of age, from all 16 Polish voivodeships (geographic regions within Poland similar to a province or state), participated in the study. The respondents gave their voluntary consent to participate and completed the questionnaire. The participants did not receive an incentive or payment for participating in the research study. The exclusion criteria were: lack of Polish citizenship, inability to communicate in Polish, impaired verbal communication, age under 18, suffering from end stage renal disease, undergoing dialysis, awaiting a kidney transplant and being after a kidney transplant.

### 2.3. Instruments 

The study used a diagnostic survey with a questionnaire technique (Computer-Assisted Web Interview). The questionnaires were distributed via the Internet by the Office for Statistical Research and Analysis (Rzeszów, Poland). The registry includes the population of adult Polish citizens. The sample obtained the representation for each voivodship by dividing the population into strata and drawing from the entire population. A self-report questionnaire was used, which included, inter alia, a sociodemographic data sheet and questions about the attitude of the respondents to live donation. Standardized tools were the Personal Values List [26] and the Polish version [22,26] of the Satisfaction With Life Scale [27]. 

The List of Personal Values consisted of two parts: the first contained 9 symbols of happiness, representing various forms of human values; the other presented 10 personal values. From the presented 9 symbols of happiness, the respondents chose 5 and assigned numbers to them successively from 5—for the most important, to 1—for the least important. According to the same key, the second group of 10 values was described. The reliability of the tool, checked by the test-retest method, was 0.78 and 0.76 of the Personal Values List at an interval of two weeks, and after six weeks it was 0.72 and 0.62, which indicates a satisfactory stability of the method [26].

The Satisfaction With Life Scale included five statements in which the respondent assessed the extent to which each of them relates to their life so far. Responses were measured using a 7—point Likert scale: 7—I strongly agree, 6—I agree, 5—I tend to agree, 4—I neither agree nor disagree, 3—I tend to disagree, 2—I disagree, 1—I strongly disagree. The result of the measurement was a general indicator of the feeling of satisfaction with life ranging from 5 to 35 points (scores of 20 are considered neutral). The instrument presents good psychometrical properties. Internal consistency measured with Cronbach’s alpha was 0.86. The stability of the results determined by the test-retest method was satisfactory (0.85–0.93 at three-week intervals; 0.87–0.88 at six-week intervals; and 0.86 at nine-week intervals) [22].

### 2.4. Outcomes 

The primary outcomes described the attitudes toward living kidney donation. The secondary outcomes reported on personal values and life satisfaction.

### 2.5. Statistical Methods 

The structure of the sample was selected by the stratified sampling method according to the representation in the population, in terms of sex, age and size of the place of residence. The minimum sample size to estimate the true percentage of the population with the required margin of error (3%) and confidence level (95%) for this study was 889 [28]. The distribution of quantitative variables, as assessed by the Shapiro-Wilk test, deviated from the normal distribution; therefore, these variables were presented as medians (Me), upper and lower quartiles (Q_25_; Q_75_), while categorical variables were presented as absolute numbers and percentages. The Mann–Whitney U test was used to compare differences between two independent groups. Inter group differences between categorical variables were evaluated with chi^2^ test. The logistic regression model was used to assess the relationship between sociodemographic factors, personal values, the total score of life satisfaction and the willingness to donate a kidney to another human. First, a logistic univariate analysis was performed to select statistically significant predictors (*p* < 0.05). Then, significant predictors were introduced into the multivariate model. The model building process was carried out by means of backward stepwise regression, using a validation mechanism based on a v-fold cross-validation. Standard measures of goodness of fit were used to evaluate the model. Logistic regression results are presented as regression coefficient, odds ratio, and confidence interval. The multivariable linear regression model was used to find the relation between sociodemographic factors and the total score of life satisfaction. Standardized regression coefficients and 95% confidence intervals (CI) were calculated for each predictor in model. Threshold of statistical significance for all tests was set at *p* =0.05. Statistical calculations were performed in STATISTICA v.13.3 (TIBCO Software Inc. (2017), Kraków, Poland). 

## 3. Results 

### 3.1. Sociodemographic Factors 

The sociodemographic data of the respondents are presented in Table 1. The final analysis covered the data of 497 women (52.1%) and 456 men (47.8%), which constituted 96% response rate. The median age of the participants in the study was 41 years. There were no significant differences in age between women and men (40 [30; 60] vs. 42 [30; 61]; Z = −1.10; *p* = 0.27). The respondents were mostly city dwellers (78.9%). About 30% of the respondents had university education, while high school graduates were 47.5% of respondents (*n* = 453), vocational education: 15.4% (*n* = 147), and primary school: 7.7% (*n* = 73). 71.5% of the participants had a stable income source (were employed/ pensioner/retired). Most of the respondents were married (64.8%), had children (60.6%) and siblings (83.6%). 70.4% of the respondents declared themselves as Catholic, while the rest declared as non-believers.

### 3.2. Willingness to Became a Living Kidney Donor

The willingness to donate a kidney to another human was declared by 755 people, which constituted 79.2% of all respondents. Among this group (*n* = 755; 100%), the largest percentage of respondents would give their kidney to a child (*n* = 677; 89.7%) and a husband / wife (*n* = 532; 70.5%), then to siblings (*n* = 478; 63.3%), a parent (*n* = 459; 60.8%), another family member (*n* = 246; 32.6%). 65.2% of participants would decide to donate a kidney to an unrelated but closely, emotionally related person (i.e., partner, *n* = 260, 34.4%; friend, *n* = 232, 30.7%), and 10.5% (*n* = 100) to a stranger. People who would donate a kidney to a stranger more often supported the legalization of this type of donation in Poland than those who would not (*n* = 84; 84% vs. *n* = 506; 59.3%; χ2 = 23.12, <0.0001), and believed that donating a kidney to a stranger is something positive (*n* = 94; 94% vs. *n* = 438; 51.3%; χ2 = 60.03, <0.0001). It is worth noting that the number of respondents who knew someone on a transplant waiting list or who had a kidney transplant was similar in the group of people considering donating their kidneys to a stranger and not declaring such willingness (*n* = 13, 13% vs. *n* = 112, 13.1%; χ2 = 0.001, *p* = 0.971).

### 3.3. List of Personal Values

The list of personal values is presented in Table 2. The five most important symbols of happiness chosen by the respondents were: good health (Me = 5), successful family life (Me = 4), being needed by other people (Me = 3), doing your favorite job, having fun (Me = 2), and good material conditions (Me = 1). The most important category of personal value for the respondents was good health, physical and mental fitness (Me = 5), then: love and friendship (Me = 4), knowledge and wisdom (Me = 3), intelligence, mental acuity (Me = 2) and joy, contentment (Me = 1). 

### 3.4. Life Satisfaction

The satisfaction with life index is shown in Supplement 1. Median score of Satisfaction with Life Scale was 20 [16; 24]. Linear regression showed that not having children (ß = −0.15; Cl95%: −0.21−0.09) and having university education (ß = −0.15; Cl95%: −0.21−0.08) reduced life satisfaction. However, it should be emphasized that the hereby proposed regression model explained no more than 4% of variance in satisfaction of life (R2 = 0.04; df model = 2; F = 21.17; *p* < 0.0001). Significantly higher level of satisfaction with life was reported by people who declared their willingness to donate a kidney to another human being compared to the respondents who did not express such a willingness (Me = 20 [16; 24] vs. 19 [16; 23]; Z = 2.25; *p* = 0.024). On the other hand, people who would like to donate their kidneys to a stranger obtained significantly less points than those who did not declare such will (18.5 [13; 23] vs. 20 [16; 24]; Z = 2.62; *p* = 0.009). In this group less people had children (*n* = 44, 44% vs. *n* = 534, 62.6%; χ2 = 23.12, *p* < 0.0001).

### 3.5. Assessment of Factors Determining Kidney Donation Willingness 

Voluntary donation of a kidney to another human being i.e., family, friends, stranger (Table 3) was more often declared by women (OR = 1.21; Cl95%: 1.03−1.42), for whom the most important symbol of happiness was a life full of adventures, travels (OR = 1.39; Cl95%: 1.06−1.82) and the most important personal value was goodness and tenderness (OR = 1.21; Cl95%: 1.05−1.40). Total scores of The Satisfaction with Life Scale correlated positively with the willingness to voluntarily donate a kidney (OR = 1.03; Cl95%: 1.003−1.06), while age correlated negatively (OR = 0.99; Cl95%: 0.98−0.99). The median age of respondents who would like to donate a kidney to another person was 8 years lower than the median age of those who would not want to (40 [29; 58] vs. 48 [33; 65]; Z = 3.17; *p* = 0.001).

### 3.6. Assessment of Factors Determining Willingness to Donate a Kidney to a Stranger

Factors determining donation of a kidney to a stranger are presented in Table 4. Respondents, for whom sense of humor and witticism were personal values (OR = 1.45; Cl95%: 1.12−1.89), who supported the legalization of kidney donation to a stranger in Poland (OR = 1.72; Cl95%: 1.28−2.32), and those who considered selfless donation of a kidney to a stranger was something positive (OR = 3.85; Cl95%: 2.51−5.92), would more likely donate a kidney to a stranger. The total score of the Satisfaction with Life Scale and age correlated negatively with the willingness to voluntarily donate a kidney. Median age of respondents who would like to donate a kidney to a stranger was significantly lower than the median age of people who did not express such will (35 [24; 46] vs. 43 [31; 62]; Z = 5.17; *p* < 0.0001).

## 4. Discussion

The results of the study showed that the vast majority of adult Poles consider donating a kidney to another person when the need arises. Kidney donation is more of an acceptable practice in the treatment of chronic renal failure for women than for men. In addition, kidney donation is supported by people for whom the most important symbol of happiness is a life full of adventures, journeys and the most important personal values are goodness and tenderness. Support also depends on age and level of life satisfaction.

As a result, we have found that 79% of Poles would be willing to donate their kidney. The respondents, as in the study by Rios Zambudio et al. [29] declared their willingness to donate the kidney primarily to relatives. In the case of unrelated people, the percentage of potential living donors was significantly lower. Almost every third respondent (31%) would give a kidney to a friend, and every tenth (10.5%) declared that they could donate a kidney to a stranger. The results of the study by Spital et al. [30], are more optimistic when analyzing donation to an unrelated person: 76% of respondents would probably give a kidney to a close friend with kidney failure, and 24% said they would even give a kidney to a stranger. Living kidney donation from unrelated donors is of increasing importance, especially in the context of research by Al. Ammary et al. [4], which shows an overall decrease in the number of related donors in the years 2017−2019. Among 19,103 American donors, relatives accounted for 41%, 45% of the transplants involved unrelated people, and 14% donated a kidney for paired donation. It should be noted that paired kidney donation is a valuable tool to overcome immune barriers to living donation and that the implementation of international kidney donation registries can be a useful strategy to facilitate transplantation [31].

Our study found that kidney donation is more often an acceptable practice in the treatment of chronic renal failure for women than for men. Similar results are presented in a study conducted among the inhabitants of Japan by Okita et al. [16]. The aforementioned authors noticed that positive attitudes towards living donation correlated with gender—women showed more positive attitudes towards donation than men. On the other hand, in the Latin American community living in Spain, men were more sympathetic to living donation than women [32]. In Singapore, gender was irrelevant to the willingness to donate [33]. However, it is worth mentioning that the willingness to donate a kidney to another person is not equivalent to becoming a donor. On the other hand, data from the Orthodox Jewish community indicate, that kidney donors were mostly men [11,34]. Study by Maple et al. [35], Faber et al. [14] and Al. Ammary et al. [4] also show the majority of donors to be male. The reasons for the gender differences in the percentage of kidney donors remain complex, poorly understood and can have. medical, psychological, sociological or economic background [36].

The findings of this study indicate that ‘’good health”, followed by “successful family life”, were the highest rated symbols of happiness among the studied Polish citizens, whereas “love and friendship” and “good health, physical and mental fitness” were the highest ranked personal values. Similarly, in Nowicki et al. [37] the same values were found to be the most important for Polish women. However, our study showed, that living kidney donation was supported by people for whom the most important symbol of happiness was a ‘’life full of adventures, travels’’ and ‘’sense of humor and witticism’’ (in the case of the willingness to give the kidney to a stranger), and the most important value was ‘’goodness and tenderness’’. Most studies on donor motivation show that the reason why they decide to donate, is to extend the life of recipients and improve their quality of life [38,39,40,41,42]. For people surveyed in Israel by Kurleto et al. [34], the motive for donating a kidney to a stranger was a strong willingness to help, and the will to do good. Helping another person was considered the most important value by over 75% of the donors [34] Willingness to help others was also an important reason for donating a kidney for UK, Swedish and US citizens [10,35,43].

It is worth noting that the support for live kidney donation also depends on age. A study by Yan et al. [18] among Chinese citizens, suggests that willingness to be living organ donors was positively correlated with age. These results are different from our study, which showed a negative correlation between the discussed variables. Respondents aged 35−40 were more willing to donate their kidneys, including donating to a stranger, than the older ones. This thesis is supported by the results of the study by Ammary et al. [4]. The researchers, analyzing the data of over 35,000 kidney donors in the years 2014−2019 in the USA, showed that the highest percentage of donors were people aged 35−49. In a study conducted in France however, no statistically significant differences were observed [44].

According to our study, the level of satisfaction with life can be a factor motivating the donation of a kidney to a person in need. In our study, a higher level of satisfaction was associated with a greater willingness of the respondents to donate a kidney. A study by Bieniasz et al. suggests [45], based on the observation of 40 Polish kidney donors lasting an average of 5 years, that the performed live kidney donation had a positive impact on the quality of life of the donors. Only those donors, who lost their recipient as a result of death were characterized by a lower level of satisfaction, but no donor regretted their decision to donate a kidney.

## 5. Strengths and Limitations

The strength of the study is the large sample size representing the whole Polish population including age, gender, education, or employment status. According to data by GUS (Polish Central Statistical Office) from the year 2020 [46], the median age for the Polish population was 41.7 years. Women accounted for 51.6% of society, and the percentage of employed, retired, and pensioners was 79.1%. Available data from 2018 shows that 27% of the population declared to have a university education [47]. Strengths of this study also include the use of validated questionnaires.

The limitations are: including only Polish citizens (however, 99.2% of Poland’s residents are Polish nationals [48]), risk of the selection bias in which the respondents chose to participate because they were interested in the topic of kidney donation. The fact that the majority of respondents were city dwellers (78.9%) may have affected the types of the answers [46].

## 6. Conclusions

In summary, our study demonstrates that respondents who declare their willingness to be a living kidney donor are mainly female, for which the most important symbol of happiness is a ‘’life full of adventures and travel’’, and the most important values are ‘’personal goodness and tenderness’’. The desire to donate a kidney to another person decreases with age and grows with life satisfaction. 

## 7. Implications for Clinical Practice

While a recipient with renal failure clearly benefits from living donation, transplant is not a medical benefit to the donor. The long-term health risks to adults who donate kidneys are also not fully understood. Based on the meta-analysis by O’Keeffe et al. [49], it can be assumed, that there is no evidence suggesting a higher risk of mortality, cardiovascular disease, hypertension, type 2 diabetes or adverse psychosocial effects (depression, anxiety, stress, lower quality relationships) among living kidney donors, compared to the general population. The absolute risk for end-stage renal disease and preeclampsia among female donors is also low. 

Therefore, in order to increase the number of living kidney donation, and thus save lives, it is important to promote living kidney donation among the Polish community, paying close attention to conducting reliable education in this field. Living donor candidates should be fully informed about the perioperative and long-term risks before deciding to donate a kidney [50]. Medical personnel should play an important role in building public awareness and increasing public support for transplantation as an effective method of treatment. The message regarding live kidney donation should be addressed to potential donors in a clear and consistent manner, should be publicly displayed and engage the recipient [6].

According to this study, the perception of happiness symbols and the system of personal values may determine the willingness to donate a kidney to another person. Knowledge about the individual’s value system can help create educational activities in such a way that organ donation will be viewed by society as a fundamental human responsibility.

## Figures and Tables

**Table 1:** Sociodemographic factors (*n* = 953).

Variable	
Age (years, Me, Q_25_; Q_75_)	41 [30; 60]
Sex	
Female	497 (52.1%)
Male	456 (47.8%)
Marital status: married	618 (64.8%)
Children—yes	578 (60.6%)
Siblings—yes	798 (83.6%)
Education	
University	280 (29.3%)
Other (High school/Vocational education/Primary school)	673 (70.5%)
Employment status: employed/pensioner/retired	682 (71.5%)
Place of residence	
City	753 (78.9%)
Village	200 (21.0%)
Religious self-identity: Catholic	672 (70.4%)

Me = median, Q_25_; Q_75_ = upper and lower quartile. Categorical variables were presented as absolute numbers and percentages.

**Table 2:** The list of personal values (*n* = 953).

Symbols of Happiness	Me [Q25; Q75]	Personal Value	Me [Q25; Q75]
Good health	5 [5; 5]	Good health, physical and mental fitness	5 [4; 5]
Successful family life	4 [4; 4]	Love and friendship	4 [4; 4]
Being needed by other people	3 [2; 3]	Knowledge and wisdom	3 [1; 3]
Doing your favorite job, having fun	2 [1; 2]	Intelligence, mental acuity	2 [1; 2]
Good material conditions	1 [0; 2]	Joy, contentment	1 [0; 2]
Scientific and professional success	0 [0; 0]	Goodness, tenderness	0 [0; 0]
Having a lot of friends	0 [0; 0]	Courage, decisiveness	0 [0; 0]
A life full of adventures, travels	0 [0; 0]	Sense of humor, witticism	0 [0; 0]
Fame, popularity	0 [0; 0]	Nice look, appearance	0 [0; 0]
		Wealth, possession	0 [0; 0]

Me = median, Q_25_; Q_75_ = upper and lower quartile.

**Table 3:** Factors determining donation of a kidney to another human being (multivariate model of logistic regression).

Variables	B	SE (B)	Wald Test	Df	P	OR (Cl95%)
Age	−0.01	0.005	6.58	1	0.010	0.99 (0.98−0.99)
The total score of the Satisfaction with Life Scale	0.03	0.01	4.61	1	0.032	1.03 (1.003−1.06)
Gender ^female vs. male^	0.19	0.08	5.23	1	0.022	1.21 (1.03−1.42)
Personal value: goodness and tenderness	0.19	0.07	6.95	1	0.008	1.21(1.05−1.40)
Symbol of happiness: a life full of adventures, travels	0.33	0.14	5.75	1	0.017	1.39 (1.06−1.82)

B = regression coefficient; SE = Standard error; df = Degrees of freedom; OR = Odds ratio; CI = Confidence interval; Logistic regression—multivariate model; R2 Nagelkerka = 0.061; Hosmer Lemeshow = 8.853; *p* = 0.354.

**Table 4:** Factors determining kidney donation willingness to a stranger (multivariate model of logistic regression).

Variables	B	SE (B)	Wald Test	Df	P	OR (Cl95%)
Age	−0.04	0.01	26.77	1	<0.001	0.96 (0.95−0.98)
The total score of the Satisfaction with Life Scale	−0.05	0.02	6.84	1	0.009	0.95 (0.92−0.99)
Personal value: a sense of humor/witticism	0.39	0.13	8.54	1	0.003	1.48 (1.14−1.92)
Support for legalization of unspecified kidney donation in Poland	0.51	0.15	11.53	1	0.001	1.66 (1.24−2.23)
Belief, that selfless donation of a kidney to a stranger is something positive	1.34	0.22	37.75	1	<0.001	3.81 (2.49−5.83)

B = regression coefficient; SE = Standard error; df = Degrees of freedom; OR = Odds ratio; CI = Confidence interval; Logistic regression—multivariate model; R2 Nagelkerka = 0.286; Hosmer Lemeshow = 5.036, *p* = 0.753.

## Data Availability

A dataset will be made available upon request to the corresponding authors one year after the publication of this study. The request must include a statistical analysis plan.
